# Supplementary material for: High-Throughput Short Sequence Typing Schemes for Pseudomonas aeruginosa and Stenotrophomonas maltophilia Pure Culture and Environmental DNA
Source: Microorganisms. 2023 Dec 27;12(1):48. doi: 10.3390/microorganisms12010048 (PMC10819370; doi:10.3390/microorganisms12010048)
Supplement: Supplementary file 1 [file microorganisms-12-00048-s001.zip › Bourdin et al, 2023 - Supplemental Figures_HiSST_Schemes_St+Pa.pdf]

Bourdin et al., 2023 (HiSST Schemes for *P. aeruginosa* and *S. maltophilia*): Supplemental figures

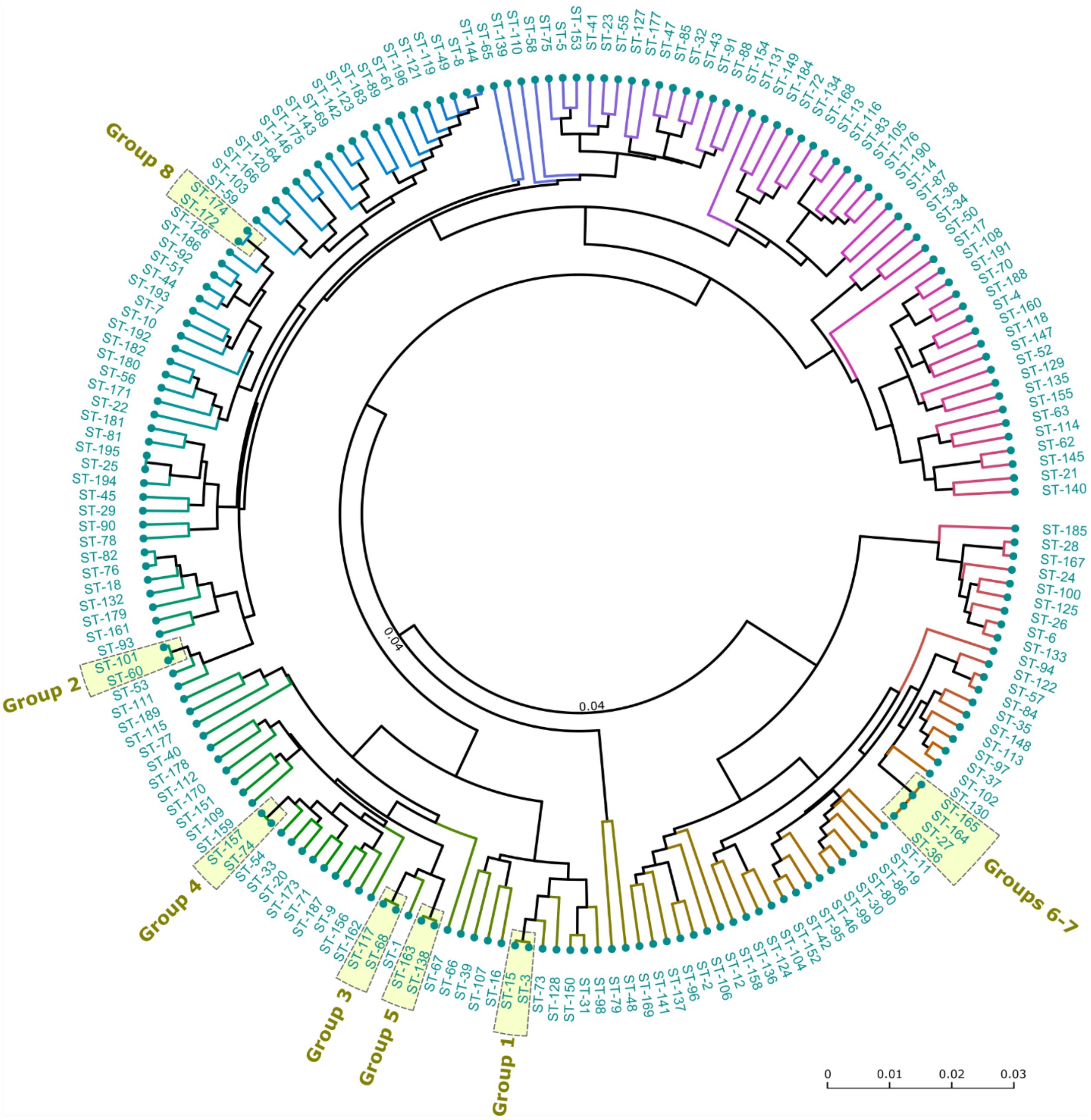

Figure S1: UPGMA tree of 196 HiSST sequence types (STs). The UPGMA tree was prepared using 196 STs that combined SST from four loci of HiSST scheme for *P. aeruginosa*, based on 513 *P. aeruginosa* genomes (Table S2A). Yellow rectangles with dash lines indicate clonal complex categorised by the eBURST programme.

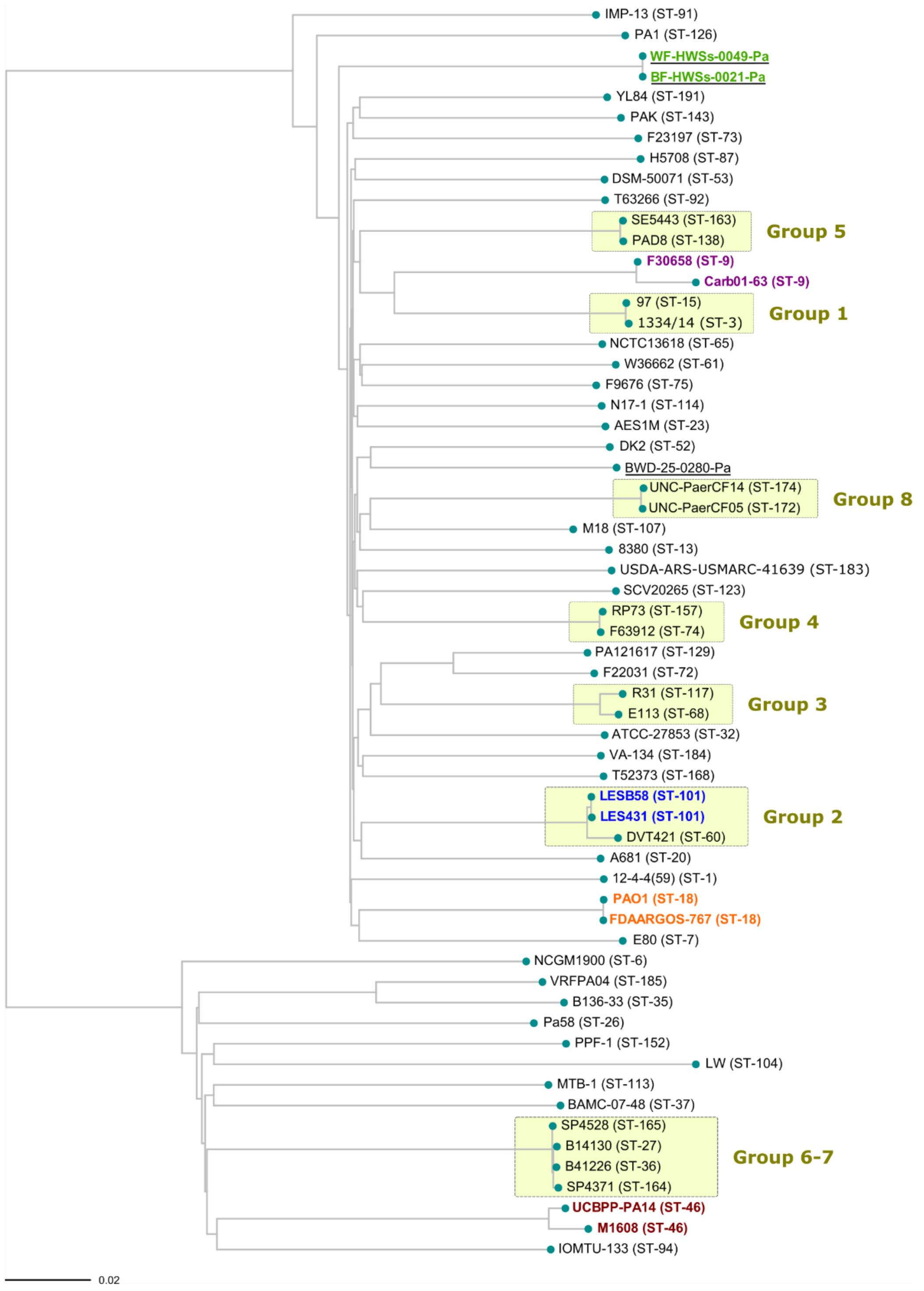

Figure S2: Haplotype tree illustrating the genetic relationships among 63 *P. aeruginosa* strains. The analysis includes all strains used in the development of the HiSST scheme, strains representing clonal complexes identified by eBURST, and three strains isolated in this study (the strain names are underlined). The tree was constructed based on a concatenation of 97,944 SNPs. SNiPlay was utilized to generate the haplotype network tree. Yellow rectangles with dashed lines

indicate clonal complexes categorized by the eBURST program. Identical STs are represented by bold colored labels.

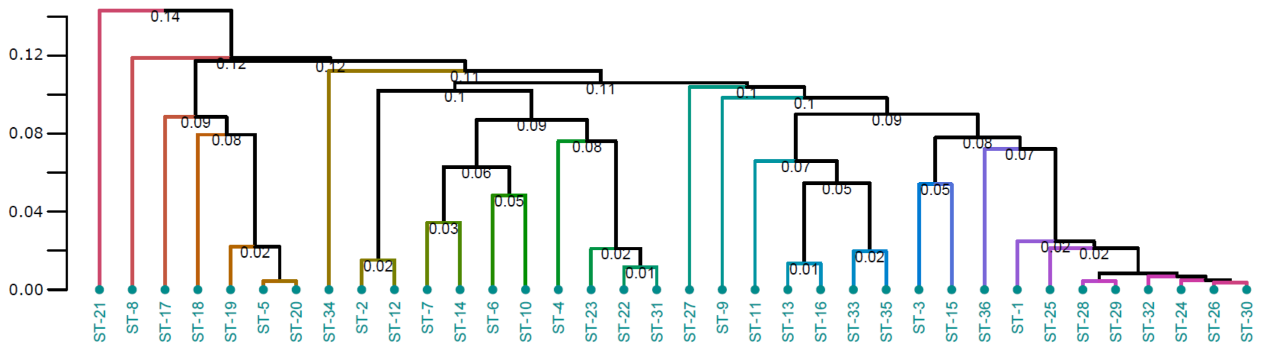

Figure S3: UPGMA tree of 36 HiSST sequence types (STs). The UPGMA tree was prepared using 36 STs that combined SST from four locus of HiSST scheme for *S. maltophilia*. None clonal complex was categorised by the eBURST programme.

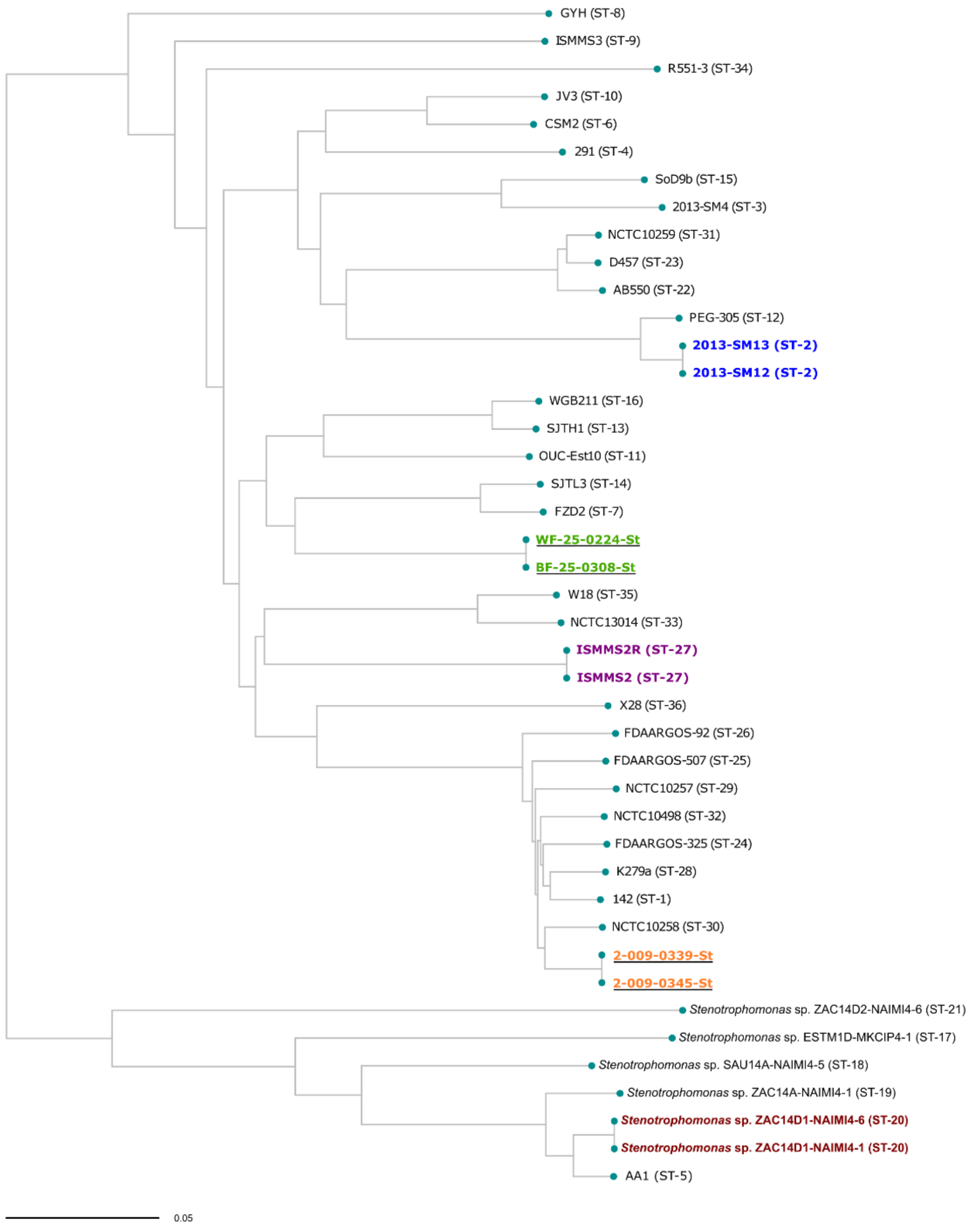

Figure S4: Haplotype tree illustrating the genetic relationships among 43 *S. maltophilia* strains. The analysis includes all complete genomes available on NCBI during the development of the HiSST scheme database, and four strains isolated in this study (the strain names are underlined). The tree was constructed based on a concatenation of 186,435 SNPs. SNiPlay was utilized to generate the haplotype network tree. Yellow rectangles with dashed lines indicate clonal complexes categorized by the eBURST program. Identical STs are represented by bold colored labels.

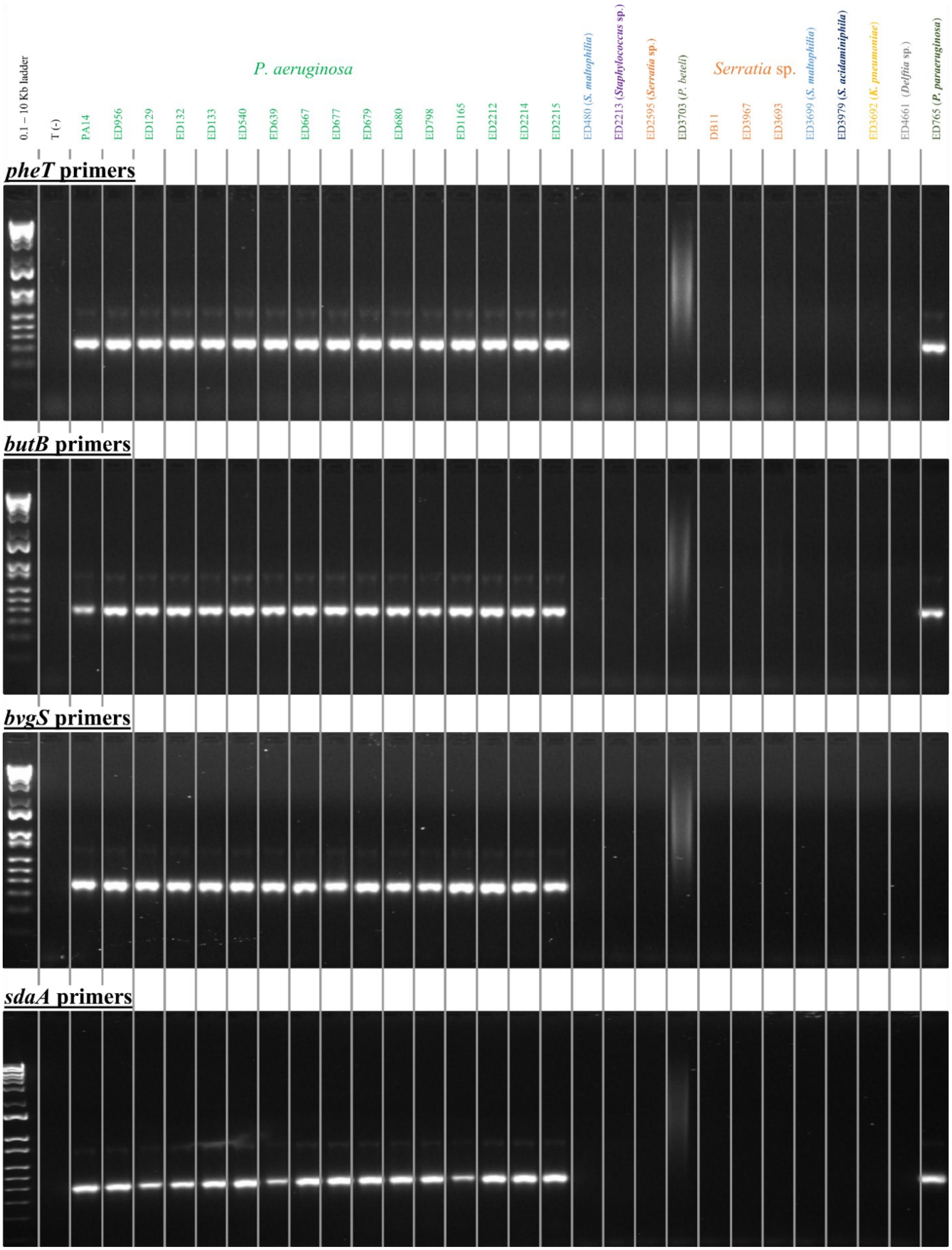

Figure S5: **In vitro tests and validation of primers designed for HiSST schemes of *Pseudomonas aeruginosa*.**

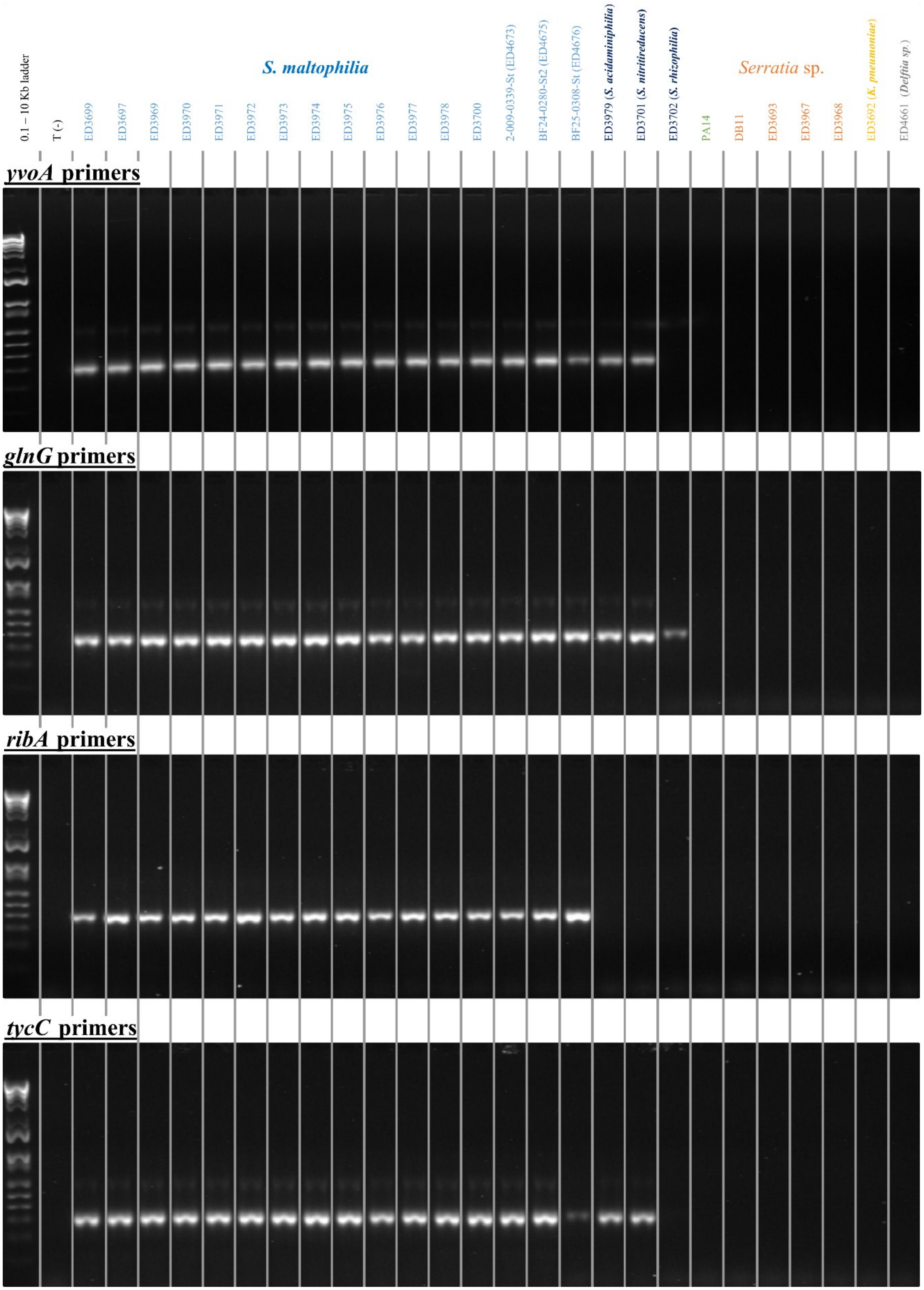

Figure S6: **In vitro tests and validation of primers designed for HiSST schemes of *Stenotrophomonas maltophilia*.**
